# Insight into Molecular Mechanism for Activin A-Induced Bone Morphogenetic Protein Signaling

**DOI:** 10.3390/ijms21186498

**Published:** 2020-09-05

**Authors:** Chen Xie, Wenjuan Jiang, Jerome J. Lacroix, Yun Luo, Jijun Hao

**Affiliations:** 1 College of Veterinary Medicine, Western University of Health Sciences, Pomona, CA 91766, USA; cxie@westernu.edu; 2 College of Pharmacy, Western University of Health Sciences, Pomona, CA 91766, USA; sibylaries@gmail.com; 3 Graduate College of Biomedical Sciences, Western University of Health Sciences, Pomona, CA 91766, USA; jlacroix@westernu.edu

**Keywords:** BMP, activin A, FKBP12, ALK2, Fibrodysplasia Ossificans Progressiva

## Abstract

Activins transduce the TGF-β pathway through a heteromeric signaling complex consisting of type I and type II receptors, and activins also inhibit bone morphogenetic protein (BMP) signaling mediated by type I receptor ALK2. Recent studies indicated that activin A cross-activates the BMP pathway through ALK2^R206H^, a mutation associated with Fibrodysplasia Ossificans Progressiva (FOP). How activin A inhibits ALK2WT-mediated BMP signaling but activates ALK2^R206H^-mediated BMP signaling is not well understood, and here we offer some insights into its molecular mechanism. We first demonstrated that among four BMP type I receptors, ALK2 is the only subtype able to mediate the activin A-induced BMP signaling upon the dissociation of FKBP12. We further showed that BMP4 does not cross-signal TGF-β pathway upon FKBP12 inhibition. In addition, although the roles of type II receptors in the ligand-independent BMP signaling activated by FOP-associated mutant ALK2 have been reported, their roles in activin A-induced BMP signaling remains unclear. We demonstrated in this study that the known type II BMP receptors contribute to activin A-induced BMP signaling through their kinase activity. Together, the current study provided important mechanistic insights at the molecular level into further understanding physiological and pathophysiological BMP signaling.

## 1. Introduction

**Figure 1:**
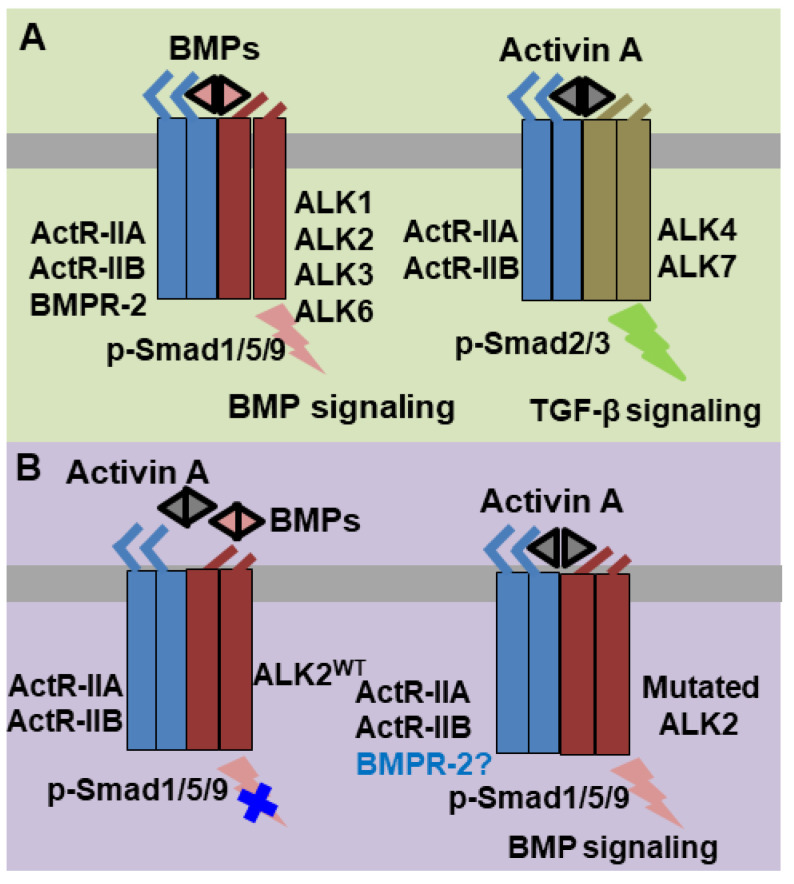
Scheme of physiologic bone morphogenetic protein (BMP) and activin A signaling pathways and abnormal activin A-induced BMP signaling in Fibrodysplasia Ossificans Progressiva (FOP). (**A**) BMPs and activin A normally assemble and bind to a heterotetramer complex consisting of a type II receptor homodimer (BMPR-II/ActR-IIA/ActR-IIB for BMPs and ActR-IIA/ActR-IIB for activin A) and a type I receptor homodimer (ALK1/2/3/6 for BMPs and ALK4/7 for activin A) to transduce signaling via Smad phosphorylation (*p*-Smad1/5/9 for BMPs and *p*-Smad2/3 for activin A). (**B**) Under normal circumstances, activin A competitively counteracts BMPs that induce BMP signaling through ALK2. However, in FOP, activin A can sensitize the mutated ALK2 to phosphorylate Smad1/5/9 for abnormal BMP signaling transduction.

Bone morphogenetic proteins (BMPs) and activins belong to the Transforming Growth Factor β (TGF-β) superfamily [1]. Their biological activities were determined by the binding to the extracellular domain of serine-threonine kinase receptors and downstream R-Smad [1,2]. Both extracellular BMPs and activins typically induce an assembly of heteromeric complexes consisting of type I and type II serine-threonine kinase receptors. This heteromerization enables type II receptors to phosphorylate type I receptors, which, in turn, phosphorylate specific R-Smad substrates to activate distinct signaling pathways. Under physiological conditions, extracellular BMPs utilize BMP Receptor type II (BMPR2) or Activin Receptors type II (ActR-IIA and ActR-IIB) as type II receptors in conjunction with Activin-Like Kinase receptors 1/2/3/6 (ALK1/2/3/6) as BMP type I receptors. Downstream BMP signaling is further relayed by the phosphorylated Smad1/5/9 proteins (Figure 1A). In contrast, activin ligands mediate TGF-β signaling by using ActR-IIA/ActR-IIB as type II receptors and ALK4/7 as type I receptors followed by the downstream phosphorylated Smad2/3 as intracellular signal transducers (Figure 1A).

Abnormal BMP signaling activation can lead to pathological ectopic ossification such as Fibrodysplasia Ossificans Progressiva (FOP) and the dominant mutation R206H in ALK2 is responsible for over 95% patients with FOP [3,4,5,6,7,8,9,10]. Initial studies indicated that ALK2^R206H^ produces basal leaky BMP signaling in the absence of BMP ligands and elicits abnormally elevated BMP signaling in the presence of BMP ligands [6,11,12,13,14,15,16,17]. However, recent findings have suggested that activin A abnormally activates BMP signaling through ALK2^R206H^ in FOP [18,19,20,21] (Figure 1B). This finding was very interesting since activin A normally transduces TGF-β signaling through ALK4/7 and acts as a competitive inhibitor of BMP signaling mediated by wild-type ALK2 (ALK2^WT^) [20]. How activin A inhibits ALK2^WT^-mediated BMP signaling, but activates ALK2^R206H^-mediated BMP signaling is not well known.

A possible mechanism may involve the ubiquitous repressor protein FKBP12 in TGF-β superfamily signaling. In the absence of ligands, FKBP12 is a negative signaling regulator which binds to a glycine-serine rich (GS) domain of type I receptors and locks the kinase catalytic domain of type I receptors in an inactivate conformation [22]. Previous studies have shown that the FKBP12 dissociation from the ALK2 resulting from the R206H mutation is critical for BMP signaling activation, in both basal leaky and hyper-responsive BMP signaling models for FOP [12,15,16]. Our recent protein–protein dynamical network analysis revealed that all three gain-of-function mutations (ALK2^R206H^, ALK2^Q207E^ and ALK2^Q207D^) located at the binding interface of ALK2 and FKBP12 share the same loss of interaction between FKBP12 and ALK2 (most significantly between G90 on FKBP12 and F246 on ALK2), which allosterically shift the kinase towards catalytic competent configuration [23,24]. Several groups pioneered the study of the implication of FKBP12 in the activin A-induced BMP signaling via the wild-type BMP type I receptor [18,19]. For instance, Hino et al. demonstrated that FK506 treatment enables the wild-type BMP type I receptor to respond to activin A for BMP signaling but did not specify which subtype(s) of the wild-type BMP type I receptor (ALK1/2/3/6) is involved in this reaction [18]. In addition, Hatsell et al. showed that FKBP12 inhibitor FK506 treatment fails to enable ALK2^WT^ for mediating the activin A-induced BMP signaling [19]. In addition, previous studies demonstrated the biological roles of type II receptors in the ligand-independent BMP signaling activated by the mutated FOP ALK2 [14,25]. However, in the activin A-induced BMP signaling, the function of these type II receptors has not yet been reported. Here, we investigated the roles of FKBP12 and BMP type II receptors in the activin A-induced BMP signaling, and further studied the specificity of type I receptors in mediating activin A-induced BMP signaling and the abilities of BMP ligands to cross-signal the TGF-β pathway.

## 2. Results

### 2.1. FKBP12 Dissociation Enables ALK2^WT^ to Mediate Activin A-Induced BMP Signaling

**Figure 2:**
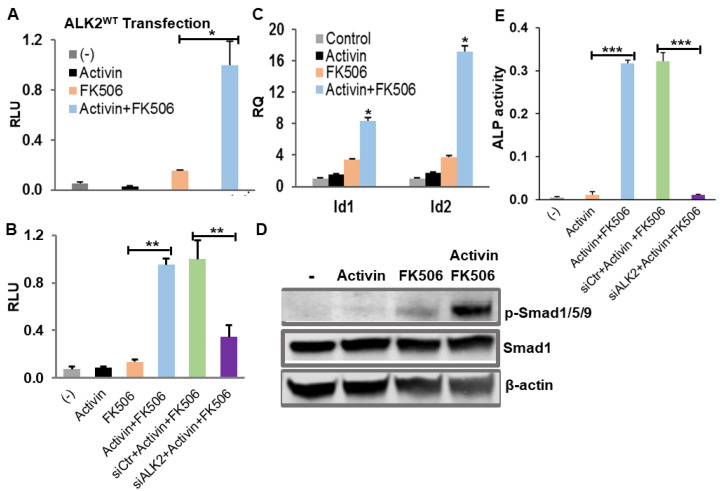
FKBP12 dissociation by FK506 enables ALK2^WT^ to mediate the activin A-induced BMP signaling. 1µM FK506 treatment significantly induced BMP signaling in the presence of 100 ng/mL activin A in the stably transfected BMP-responsive C2C12BRA cells (**A**) with ALK2^WT^ transfection and (**B**) without ALK2^WT^ transfection. Knockdown ALK2 by siRNA ALK2 (siALK2) resulted in a significant decrease in the activin A-induced BMP signaling in C2C12BRA cells (siCtr: scrambled siRNA). (**C**) RT-PCR showed that the mRNA expression levels of the BMP signaling target genes, Id1 and Id2, were dramatically elevated by the treatments of FK506 and Activin A. (**D**) Western blotting result indicated that the treatments of FK506 and activin A increased the expression of the phosphorylated Smad1/5/9 (*p*-Smad1/5/9). (**E**) Alkaline phosphatase (ALP) assay results demonstrated that the treatments of FK506 and activin A significantly increased ALP activity in C2C12 cells and this ALP-induced activity is dramatically suppressed upon siRNA ALK2 knockdown. Results of luciferase assay and RT-PCR assay were represented as mean relative luciferase units (RLU)  ±  SEM and relative quantity (RQ) ± SEM (*n*  =  3, * *p* < 0.05, ** *p* < 0.01, *** *p* < 0.001).

We first tested if FKBP12 dissociation by FK506 enables ALK2^WT^ to mediate activin A-induced BMP signaling. In brief, we transiently transfected C2C12BRA cells (C2C12 cells stably transfected with the Id1 promoter-firefly luciferase reporter) with ALK2^WT^ plasmid and renilla luciferase reporter pRL-TK plasmid as internal control, followed by the treatment with FKBP12 inhibitor FK506. The luciferase assay showed that 1 µM FK506 dramatically induced BMP signaling in the ALK2^WT^- transfected C2C12BRA cells treated with activin A, and modestly activated BMP signaling in the absence of activin A, which could be attributed to the trace amount of BMPs or activins in the culture media (Figure 2A). Previous reports demonstrated that ALK2 is the only detectable BMP type I receptor subtype in the C2C12BRA cells [26], therefore we assumed that the FK506 treatment would also promote the activin A-induced BMP signaling in the C2C12BRA cells through endogenous ALK2 without ALK2 plasmid transfection. As expected, the luciferase assay showed that activin A significantly activated BMP signaling in the presence of FK506 via the endogenous receptors (Figure 2B). Furthermore, we knocked down ALK2 by siRNA in C2C12BRA cells, and the luciferase assay showed that the BMP signaling induced by the activin A was decreased by 66% (Figure 2B).

Because the luciferase assay used in the C2C12BRA cells is BMP signaling specific, and ALK4/7 do not signal BMP signaling but TGF-β signaling, our results indicate that FKBP12 dissociation by FK506 can enable ALK2^WT^ to mediate activin A-induced BMP signaling. Moreover, the activation of BMP signaling by activin A in the presence of FK506 was also confirmed by the BMP signaling target gene Id1 and Id2 expression assessed with RT-PCR as well as the phosphorylated Smad1/5/9 proteins measured by Western blotting (Figure 2C,D). In addition, C2C12 myoblasts have the ability to differentiate into the osteoblast lineage when BMP signaling is activated and alkaline phosphatase (ALP) is known as an early marker of osteoblast differentiation [16,27]. We therefore examined ALP activity in C2C12 cells with various treatment conditions. As expected, activin A treatment significantly enhanced ALP activity in the presence of FK506, and the induced-ALP activity was dramatically suppressed by siRNA ALK2 knockdown (Figure 2E).

**Figure 3:**
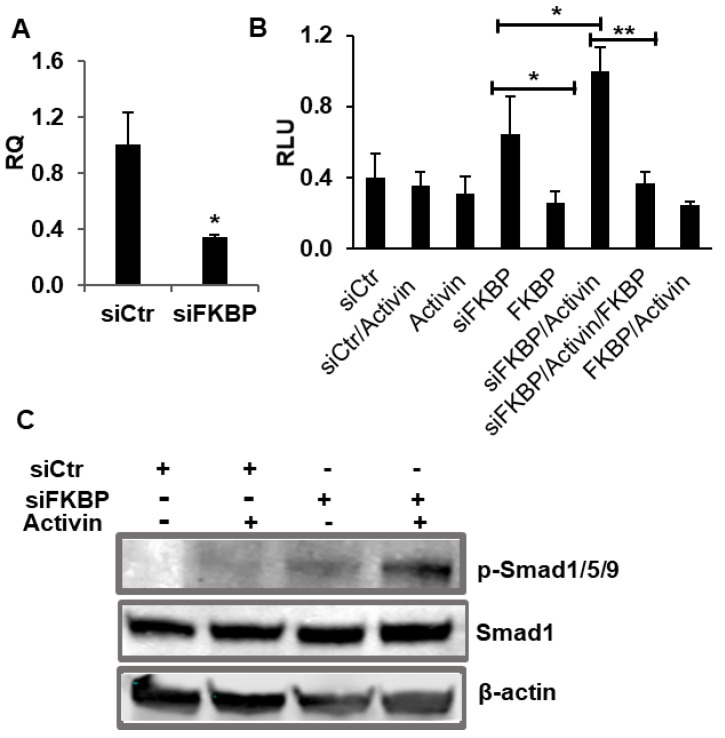
siRNA knockdown of FKBP12 enables ALK2^WT^ to mediate the activin A-induced BMP signaling in C2C12BRA cells. (**A**) Specific siRNA (siFKBP) effectively knocked down FKBP12 in the stably transfected BMP-responsive C2C12BRA cells (siCtr: scrambled siRNA). (**B**) Luciferase assay result indicated that FKBP12 knockdowns induced activin A-induced BMP signaling, whereas the overexpression of FKBP12 suppressed the activin A-induced BMP signaling in C2C12BRA cells. (**C**) Western blotting confirmed that FKBP12 knockdowns induced activin A-induced BMP signaling. Results of luciferase assay and RT-PCR assay were represented as the mean relative luciferase units (RLU)  ±  SEM and relative quantity (RQ) ± SEM (*n*  =  3, * *p* < 0.05, ** *p* < 0.01).

To rule out the possibility of the off-target effect of small molecule FK506 in the activin A-induced BMP signaling, we knocked down FKBP12 with a specific siRNA and then examined the activin A-induced BMP signaling mediated by the endogenous ALK2^WT^. Our results showed that the FKBP12-targeting siRNA effectively knocked down over 60% of FKBP12 mRNA in C2C12BRA cells (Figure 3A) and resulted in the dramatic activation of BMP signaling induced by activin A as indicated in both luciferase assay and Western blotting (Figure 3B,C). In addition, the overexpression of FKBP12 dramatically suppressed the activin A-induced BMP signaling (Figure 3B). As expected, the amplitude of BMP signaling was not significantly altered when using a scrambled siRNA as a control (Figure 3B,C).

In summary, our results of both the FK506 treatment and siRNA knockdown demonstrate that FKBP12 dissociation enables ALK2^WT^ to mediate activin A-induced BMP signaling. It is consistent with Hino’s study that the inhibition of FKBP12 can enable the wild-type BMP type I receptor (despite no specific subtype of the BMP type I receptor being identified in their study) to mediate the activin A-induced BMP signaling [18]. In contrast, Hatsell et al. concluded that FKBP12 dissociation was unable to activate ALK2^WT^ for the activin A-induced BMP signaling [19]. In their study, a range of FK506 concentrations from 10^−17^ to 10^−10^ M was used and such low concentrations may not be sufficient for FKBP12 dissociation [19]. Indeed, our experiment further confirms that FK506 in such a low dose (10^−10^ M) is unable to activate ALK2^WT^ for activin A-induced BMP signaling (Figure S1).

### 2.2. Activin A-Induced BMP Signaling Is Only Mediated by Type I Receptor ALK2

**Figure 4:**
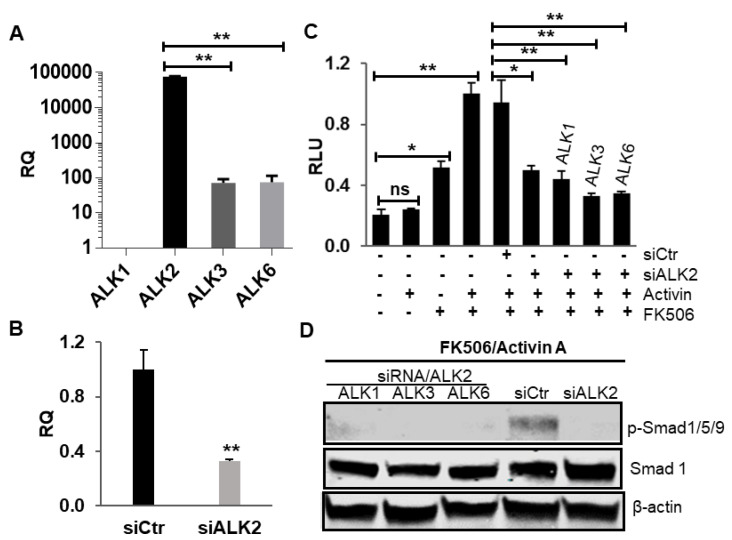
Only BMP type I receptor subtype ALK2, but not ALK1/3/6, is able to mediate activin A-induced BMP signaling upon FKBP12 dissociation. (**A**) RT-PCR confirmed that ALK2 is the only predominately expressed BMP type I receptor subtype in HepG2BRA cells and only a trace amount of ALK3 and ALK6 can be detected (note: the *y* axis is in log scale). The expression level of ALK1 was set as 1, and all the other numbers were normalized with ALK1. (**B**) The specific siRNA effectively knocked down ALK2 in HepG2BRA cells. (**C**) None of the three BMP type I receptor subtypes (ALK1/3/6) could rescue the luciferase downregulation caused by ALK2 knockdown in the presence of activin A and FK506, suggesting that ALK1/3/6 cannot signal activinA-induced BMP signaling upon FKBP12 dissociation, and (**D**) this result was confirmed by Western blotting. Results of luciferase assay and the RT-PCR assay were represented as a mean relative luciferase units (RLU)  ±  SEM and relative quantity (RQ) ± SEM (*n*  =  3, * *p* < 0.05, ** *p* < 0.01).

Since FKBP12 acts as a ubiquitous repressor for the four subtypes of the BMP type I receptor (ALK1/2/3/6), our results raised the possibility that activin A could also mediate BMP signaling via ALK1/3/6. We addressed this question by using HepG2BRA cells (i.e., HepG2 cells stably transfected with BRE-Luc construct) as these cells were previously reported to be extremely sensitive for BMP signaling and express only ALK2 as BMP type I receptor [26]. To confirm that ALK2 is the only BMP type I receptor subtype present in HepG2BRA cells, we examined all four BMP type I receptor subtype mRNA expressions by RT-PCR. Our results showed that HepG2 cells do not express ALK1 while only a trace amount of ALK3/6 mRNA was detected (Figure 4A, note: *y* axis is log scale). We next knocked down the ALK2 gene with siRNA in HepG2BRA cells and transfected them with ALK1, ALK3 or ALK6, respectively. Transfected cells were then treated with activin A, FK506 or their combination. Our RT-PCR assay confirmed that ALK2 expression was effectively knocked down by siRNA treatment (Figure 4B). In addition, our luciferase assay demonstrates that activin A-induced BMP signaling upon FK506 treatment was significantly down-regulated upon siRNA-mediated ALK2 knockdown. However, none of the three BMP type I receptor subtypes (ALK1/3/6) was able to rescue the downregulation of BMP signaling caused by ALK2 knockdown in the presence of activin A and FK506 (Figure 4C). The same result was also confirmed by Western blotting (Figure 4D), suggesting that ALK2 is the only subtype of BMP type I receptor capable of mediating activin A-induced BMP signaling upon FKBP12 dissociation. The overexpression of ALK1, ALK3 and ALK6 was confirmed by RT-PCR (Figure S2).

### 2.3. BMP Ligand Does Not Cross-Signal TGF-β Pathway upon FKBP12 Inhibition

**Figure 5:**
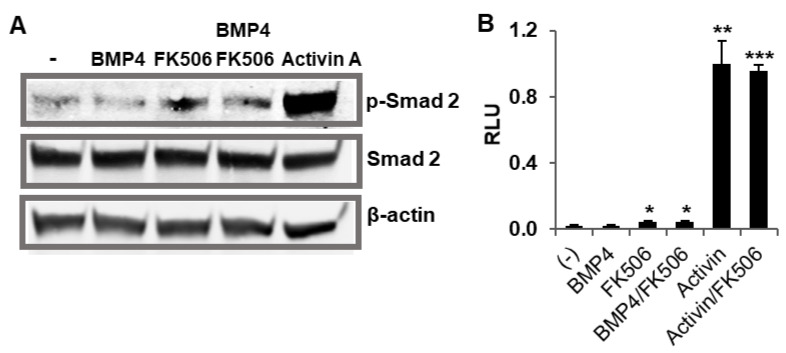
BMP4 cannot cross-signal TGF-β signaling in the absence of FKBP12. (**A**) Western blotting indicated that BMP4 did not induce TGF-β signaling in the presence of FK506 for FKBP12 dissociation, and (**B**) the same result was confirmed by the luciferase assay. Results of the luciferase assay were represented as the mean relative luciferase units (RLU)  ±  SEM, and all the *p* values were compared to the luciferase activity of the non-treatment control (*n*  =  3, * *p* < 0.05, ** *p* < 0.01,*** *p* < 0.001).

Since FKBP12 acts as an ubiquitous repressor for both BMP and TGF-β kinases and our results demonstrate a cross-talk between a TGF-β ligand (activin A) and a BMP receptor (ALK2) upon FKBP12 dissociation, we investigated whether a BMP ligand could, in turn, induce TGF-β signaling upon FKBP12 dissociation. To test this hypothesis, HEK293 cells were treated with BMP4, FK506 and their combination and the cell lysates were subjected to Western blotting. As expected, our control assays showed that TGF-β signaling was induced by activin A but not by BMP4 (Figure 5A). The FK506 treatment alone slightly elevated phosphorylated Smad2, a marker of TGF-β signaling. This moderate effect could be attributed to TGF-β ligands present in the culture medium or to the basal kinase activity of unrepressed TGF-β receptor or a combination of both (Figure 5A). However, co-treatment with BMP4 and FK506 did not further increase FK506-induced phosphorylated Smad2 levels. To confirm the above Western blotting result, we then conducted TGF-β signaling-specific luciferase assays in HEK293 cells. Briefly, the HEK293 cells were transiently transfected with plasminogen activator inhibitor-1 (PAI-1) promoter-luciferase construct [28,29], followed by treatments with BMP4, FK506 and their combination. Consistent with the Western blotting result, our luciferase assay indicates that BMP4 is unable to increase the level of TGF-β signaling, indicating that BMP ligands do not cross-signal the TGF-β pathway upon FKBP12 inhibition (Figure 5B).

### 2.4. Activin A-Induced BMP Signaling Requires Type II Receptor Kinase Activity

**Figure 6:**
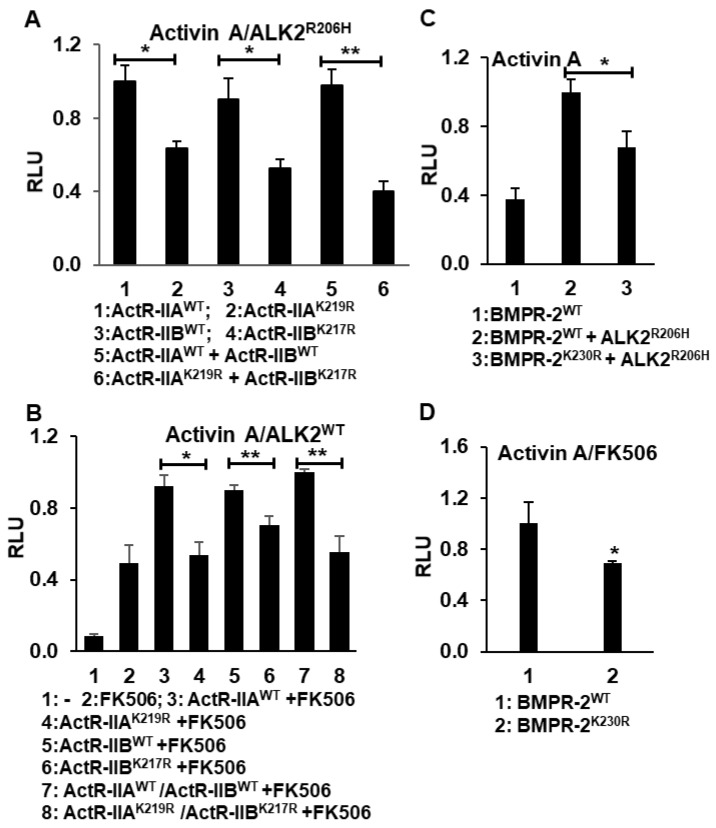
The type II receptor kinase activity is required in activin A-induced BMP signaling. (**A**) The overexpression of kinase-deficient ActR-IIA^K219R^ and ActR-IIB^K217R^ significantly downregulated activin A-induced BMP signaling via FOP ALK2^R206H^. (**B**) The overexpression of kinase-deficient ActR-IIA^K219R^ and ActR-IIB^K217R^ significantly downregulated activin A-induced BMP signaling via ALK2^WT^ upon FKBP12 dissociation in HepG2BRA cells. (**C**) The overexpression of kinase-deficient BMPR-2^K230R^ downregulated activin A-induced BMP signaling via FOP ALK2^R206H^ and (**D**) via ALK2^WT^ upon FKBP12 dissociation. All the luciferase assays were conducted in the stably transfected BMP-responsive HepG2BRA cells. Results of the luciferase assay were represented as the mean relative luciferase units (RLU)  ±  SEM (*n*  =  3, * *p* < 0.05, ** *p* < 0.01).

As aforementioned, controversial roles of the type II receptor have been reported in the BMP ligand-stimulated FOP model. One report supported an essential kinase role for the type II receptor, whereas another study argued the non-enzymatic scaffolding function of the type II receptor [14,25]. To date, it remains unknown how type II receptors contribute to pathological activin A-induced BMP signaling in FOP (i.e., via ALK2^R206H^). Since ActR-IIA and ActR-IIB are the only known type II receptors for activin A signaling, we first generated the two kinase-deficient mutants ActR-IIA^K219R^ and ActR-IIB^K217R^. Then, the following combinations of plasmids, in combination with renilla luciferase reporter pRL-TK plasmid as an internal control, were transiently transfected into HepG2BRA cells: (1) ActR-IIA^WT^/ALK2^R206H^, (2) ActR-IIA^K219R^/ALK2^R206H^, (3) ActR-IIB^WT^/ALK2^R206H^, (4) ActR-IIB^K217R^/ALK2^R206H^, (5) ActR-IIA^WT^/ActR-IIB^WT^/ALK2^R206H^ and (6) ActR-IIA^K219R^/ActR-IIB^K217R^/ALK2^R206H^. After overnight incubation with activin A, a dual luciferase assay was conducted. Our data showed that the over-expression of either kinase-deficient ActR-IIA^K219R^ or ActR-IIB^K217R^ as well as their combination significantly downregulated activin A-induced BMP signaling via ALK2^R206H^, suggesting that the kinase activity of the type II receptor is required for activin A-induced BMP signaling in FOP disease (Figure 6A).

Then, we examined the role of type II receptor kinase activity in activin A-induced BMP signaling via ALK2^WT^. Luciferase assay in HepG2BRA cells showed that BMP signaling is induced by a co-treatment with FK506 and activin and that the level of BMP signaling is further enhanced by the heterologous over-expression of ActR-IIB^WT^ or ActR-IIA^WT^ or their combination. However, this enhancement was not present when the cells were transiently transfected with kinase-deficient ActR-IIA^K219R^ and ActR-IIB^K217R^ mutants. This result demonstrates that the contribution of ActR-IIB and ActR-IIA type II receptors to activin A-induced BMP signaling depends on their kinase activity (Figure 6B).

Although the type II receptor BMPR-2 is known not to mediate TGF-β signaling in physiological conditions, it could participate in pathological activin A-induced BMP signaling. To test this hypothesis, we used the kinase-deficient BMPR-2^K230R^ mutant. The following combinations of plasmids together the with renilla luciferase reporter pRL-TK plasmid as the internal control were transiently transfected into HepG2BRA cells: (1) BMPR-2^WT^/ALK2^WT^, (2) BMPR-2^WT^/ALK2^R206H^ and (3) the kinase-deficient BMPR-2^K230R^/ALK2^R206H^. As was the case with ActR-IIB and ActR-IIA type II receptors, co-expressing BMPR-2^WT^ and ALK2^R206H^ produces a significantly higher BMP signal as compared to co-expressing BMPR-2^K230R^ and ALK2^R206H^. This shows that the BMPR-2 receptor kinase’s activity contributes to activin A-induced BMP signaling (Figure 6C). The same conclusion was obtained when measuring activin A-induced BMP signaling via ALK2^WT^ in the presence of FK506 (Figure 6D). In addition, similar experimental results were obtained from C2C12BRA cells, further supporting the important role of Type II receptor kinase activity in the activin A-induced BMP signaling (Figure S3).

## 3. Discussion

TGF-β superfamily pathways play essential roles in many cellular processes including proliferation, differentiation, migration and survival. BMPs and activins belong to the TGF-β superfamily, and they utilize different receptors and R-Smads to mediate distinct BMP and TGF-β subtype pathways, respectively. FKBP12 function as a common inhibitor for both BMP signaling and TGF-β signaling by binding to their corresponding type I receptors (ALK1/ALK2/ALK3/ALK6 for BMP signaling inhibition and ALK4/ALK7 for activin-induced TGF-β signaling inhibition). No cross-signaling for BMPs and activins were recognized until a recent discovery that activin A can induce BMP signaling via FOP ALK2 mutants such as ALK2^R206H^. It has been previously shown that FOP mutations promote the dissociation of FKBP12 from ALK2, leading to BMP signaling leakage, suggesting that FKBP12 dissociation may enable ALK2^WT^ to mediate the activin A-induced BMP signaling. In this regard, Hatsell et al. showed that the FKBP12 inhibitor FK506 treatment fails to enable ALK2^WT^ for mediating the activin A-induced BMP signaling [19]. On the other hand, Hino et al. demonstrated that FK506 treatment enables the wild-type BMP type I receptor to respond to activin A for BMP signaling but did not specify which subtype(s) of the wild-type BMP type I receptor (ALK1/2/3/6) is involved in this reaction [18]. By utilizing multiple assays including siRNA knockdown, here we demonstrated that FKBP12 dissociation enables ALK2^WT^ to mediate activin A-induced BMP signaling.

Moreover, we proved that only ALK2, but not any other three BMP type I receptor subtypes (ALK1/3/6), can mediate the activin A cross-talking BMP signaling upon FKBP12 dissociation. In addition, as FKBP12 is a common negative inhibitory factor in regulating both BMP and TGF-β signaling pathways, we investigated whether BMP ligands can also cross-signal TGF-β signaling upon FKBP12 dissociation in a similar way to the activin A activation for BMP signaling. Our data indicate that BMP ligands failed to cross-signal TGF-β signaling in the FKBP12 dissociation, suggesting other factors additional to FKBP12 dissociation may govern the ligand-specific TGF-β signaling.

In the previous ligand-independent BMP signaling of the FOP model, two groups showed conflicting results about the role of the type II receptor’s roles [14,25]. Fujimoto et al. demonstrated that the kinase activity of the type II receptors is required for the phosphorylation of Thr203 in ALK2^R206H^ for BMP signaling activation [25], whereas Bagarova et al. showed that the type II receptors play a nonenzymatic scaffolding function in the ligand-independent BMP signaling activation [14]. However, in the recently discovered activin A-induced BMP signaling in FOP, what role the type II receptors perform has to date not been reported. Our study of kinase-deficient type II receptors in this scenario demonstrates that the kinase activity of either three type II receptors, ActR-IIA or ActR-IIB, BMPR-2, are critical for the activin A-induced BMP signaling in FOP. In summary, our study provides important insight into the molecular mechanism for activin A-induced BMP signaling.

## 4. Materials and Methods

### 4.1. Cell Culture

C2C12-BRA cells (Mouse myoblast cells stably transfected with BRE-Luc construct) and HepG2-BRA (human hepatoma cell lines stably transfected with BRE-Luc construct) were obtained from Dr. Rifkin’s lab at New York University (New York, NY, USA), HEK293 cells (human embryonic kidney, ATCC) were cultured in DMEM supplemented with 10% fetal bovine serum (FBS) (Gibco, Grand Island, NY, USA) and 1% penicillin–streptomycin (Invitrogen, Carlsbad, CA, USA). Cultures were maintained in a humidified incubator at 37 °C in 5% CO_2_.

### 4.2. Plasmids and Primers

The pcDNA3.1 plasmids harboring the long form BMPR-2 and its kinase dead mutant BMPR-2^K230R^ were kindly provided by Dr. Petra Knaus at Freie University Berlin (Berlin, Germany). The following plasmids were used in the current study: pCDNA3 plasmids harboring ALK2^WT^, ALK3^WT^ and ALK6^WT^ were purchased from Addgene (#80870, #80873 and #80882). pCMV3 plasmids harboring activin A receptor type 2A (ActR2A) and human activin A receptor type 2B (ActR2B) were obtained from Sino Biological Inc. The point mutations in human ALK2 (ALK2^R206H^), human ActR2A (ActR2A^K219R^) and ActR2B (ActR2B^K217R^) were made in-house using the QuikChange II site directed mutagenesis method. The sequences for mutagenic oligonucleotides are shown in Supplementary Table S1. The mutations were confirmed by DNA sequencing.

### 4.3. Transfection

Transient transfection for overexpression was performed using Fugene HD transfection reagent (Promega, Madison, WI, USA) according to the manufacturer’s instructions. Briefly, C2C12-BRA cells or HepG2-BRA cells or HEK293 cells were seeded in 96-well plates to culture in the growth medium DMEM/10% FBS without antibiotics. After overnight culture, the cells were transfected with plasmids. For the knockdown experiments, the C2C12-BRA or HepG2-BRA cells were transfected with Fugene HD transfection reagent (Promega) using pools of FKBP12 Mouse siRNA oligo Duplex (SR401368, OriGene, Rockville, MD, USA) or ALK2 Human siRNA oligo Duplex (SR300056, OriGene) at a concentration of 20 nM, respectively. When the siRNA pools were combined, 20 nM of each 27 siRNA duplexes was used. Samples for the dual-luciferase assay or Western blot were taken 30 h post-transfection after the treatment with drugs.

### 4.4. Luciferase Reporter Assay

The cells were transfected with different kinds of plasmids along with renilla luciferase reporter pRL-TK plasmid. Five hours after transfection, the cells were starved in DMEM containing 0.5% FBS for 4 h. Then, the cells were untreated or treated with 1 μM FK506 (Cayman) or 100 ng/mL activin A (R&D) or 1 μM FK506/100 ng/mL activin A or 50 ng/mL BMP4 (Euzlab) or 50 ng/mL BMP4/1μM FK506 overnight before lysis. Luciferase activities were determined according to the dual-luciferase1reporter assay system (Promega) using the Renilla for normalization of transfection efficiency.

### 4.5. Real-Time PCR

RNA was extracted by resuspending the cells in lysis buffer and purified by filtration following the manufacturer’s protocol (GeneJET RNA Purification Kit, Thermo Scientific, Waltham, MA, USA). The first-strand cDNAs were synthesized using the High-Capacity cDNA Reverse Transcription kit (Applied Biosystems) according to the manufacturer’s instructions. Using cDNA as template, real-time (RT)-PCR reactions were carried out using the Fast Syber Green (2×) Master Mix Applied Biosystems). The reactions were performed in triplicate on a Bio-Rad CFX connected Real-Time PCR system. Human or mouse glyceraldehyde-3-phosphate dehydrogenase (GAPDH) gene was used as an internal control. The primer sets used in this study are shown in Table S1.

### 4.6. Western Blotting

Cells were lysed with RIPA buffer (Sigma) containing protein inhibitors (complete ULTRA Tablets, Roche, Basel, Switzerland) and phosphatase inhibitors (PhosSTOP, Roche, Basel, Switzerland). Samples were denatured by incubating at 95 °C for 5 min in sample buffer and separated by using SDS-PAGE (precast 8–16% gradient gels, Biorad, Hercules, CA, USA). Then, the samples were transferred to a PDVF membrane (Millipore). The membrane was blocked with Odyssey Blocking solution (Li-Cor Biosciences, Lincoln, NE, USA) for 1 h at room temperature, followed by primary antibody incubation at 4 °C overnight. The primary antibodies used in the present study included rabbit anti-*p*-Smad1/5/9 (Cell Signaling Tech, Danvers, MA, USA), rabbit anti-Smad 1(Cell Signaling Tech), mouse anti-beta actin (Cell Signaling Tech), rabbit anti-*p*-Smad 2 (Cell Signaling Tech) and rabbit anti-Smad 2 (Cell Signaling Tech). The proteins were detected by Odyssey system (Li-Cor bioscience) followed by the secondary antibodies including IRDye 680-conjugated goat anti-rabbit IgG (Li-Cor Bioscience) and IRDye 800CWconjugated goat anti-mouse IgG (Li-Cor Bioscience, Lincoln, NE, USA).

### 4.7. ALP Activity Assay

The C2C12 cells were plated at 8000 cells/well in 96-well plates and grown overnight in DMEM containing 10% FBS. siRNA ALK2 or siRNA control were transfected for some treated groups. Then, the culture medium was replaced with DMEM containing 2% FBS and the cells were untreated or treated with 100 ng/mL activin A (R&D) or 1 μM FK506/100 ng/mL activin A. After 72 h treatments, the cells were washed with PBS buffer and lysed with 50 µL lysis buffer (0.5% Triton-I00/H2O) and frozen/thawed three times to disrupt the cell membranes. The cell lysates were centrifuged for 5 min at 13,000× *g*. The supernatant was collected and the aliquots (30 µL) were subjected to an ALP activity assay at 405 nm absorbance by using the ALP activity assay kit (Bioassay Systems, Hayward, CA, USA).

### 4.8. Statistical Analysis

All values are expressed as the means ± SEM (standard error). The comparison of the means was conducted using the Student’s *t* test, and the results were considered statistically significant if the *p*-value was *<* 0.05.

## Supplementary Materials

Supplementary materials can be found at https://www.mdpi.com/1422-0067/21/18/6498/s1.

## Abbreviations

BMP	Bone Morphogenetic Protein
TGF-β	Transforming Growth Factor β
FOP	Fibrodysplasia Ossificans Progressiva
BMPR2	BMP Receptor type II
ActR-IIA	Activin Receptors type IIA
ActR-IIB	Activin Receptors type IIB
ALK1	Activin Receptor-like Kinase 1
ALK2	Activin Receptor-like Kinase 2
ALK3	Activin Receptor-like Kinase 3
ALK4	Activin Receptor-like Kinase 4
