# Supplementary material for: Insight into Molecular Mechanism for Activin A-Induced Bone Morphogenetic Protein Signaling
Source: Int J Mol Sci. 2020 Sep 5;21(18):6498. doi: 10.3390/ijms21186498 (PMC7555472; doi:10.3390/ijms21186498)

Supplementary Material

Supplementary Table 1: Oligonucleotides used in the study

Genes	Oligonucleotides
Human ALK2^{R206H}	5'-CAAAGAACAGTGGCTCACCAGATTACACTGTTG-3' (forward)<br>5'-AACAGTGTAATCTGGTGAGCCACTGTTCTTTG-3' (reverse)
Human ActRIIA^{K219R}	5'-CGAATATGTGGCTGTCAGAATATTTCCAATACAGGAC-3' (forward)<br>5'-GTCCTGTATTGGAAATATTCTGACAGCCACATATTCG-3' (reverse)
Human ActRIIB^{K217R}	5'-GAATGACTTTGTAGCTGTCAGGATCTTCCCACTCCAG-3' (forward)<br>5'-CTGGAGTGGGAAGATCCTGACAGCTACAAAGTCATTC-3'(reverse)
Human GAPDH	5'-GGTGTGAACCATGAGAAGTATGA-3' (forward)<br>5'-GTCCTTCCACGATACCAAAG-3' (reverse)
Mouse GAPDH	5'-CCCATCACCATCTTCCAGGAGC -3' (forward)<br>5'-CCAGTGAGCTTCCCGTTCAGC -3' (reverse)
Human ALK1	5'-CAACAGTCCAGAGAAGCCTAAA-3' (forward)<br>5'-CTCACACTACCTCTACCCAGATA-3' (reverse)
Human ALK2	5'-GTGACCAAGAGCCTGCATTA-3' (forward)<br>5'-TTGGGCTTCTCATCTTCCATAC-3' (reverse)
Human ALK3	5'-AGTGGGTCTGGACTACCTTTA-3' (forward)<br>5'-GCCCATCCATACTTCTCCATATC -3' (reverse)
Human ALK6	5'-CCTATACACCACAGGGCTTTAC-3' (forward)<br>5'-CGAGGTCTGGTTTCTTGTCTT-3' (reverse)
Mouse ALK2	5'-CCTGGAAGTTGGCCTTATCA -3' (forward)<br>5'-GTGGTGATGAGCCCTTCAAT -3' (reverse)
Mouse FKBP12	5'-TGTTCCGTCACTCAGCTTTG -3' (forward)<br>5'-GTTAACCCATCGCTTGTGCT-3' (reverse)
Mouse Id1	5'-AGGTGAACGTCCTGCTCTA -3' (forward)<br>5'-GATCTCCACCTTGCTCACTTT-3' (reverse)
Mouse Id2	5'-CTACTCCAAGCTCAAGGAACTG-3' (forward)<br>5'-GATCTGCAGGTCCAAGATGTAA-3' (reverse)

Supplementary Figure 1: FK506 at 10^{-10} M concentration is unable to activate ALK2^{WT} for the activin A-induced BMP signaling.

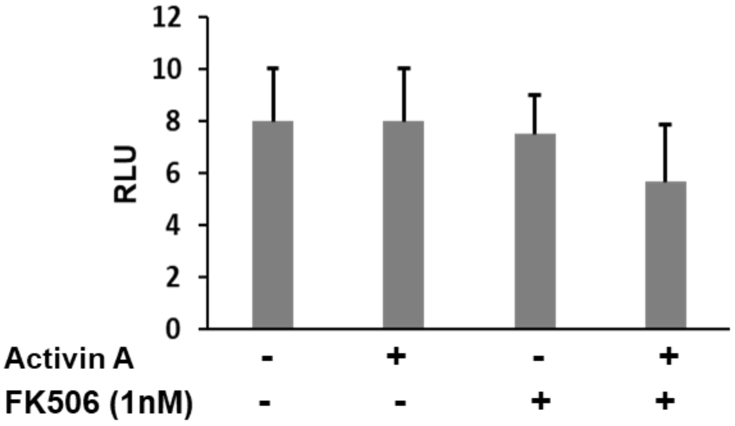

Supplementary Figure 2: Overexpression of ALK1, ALK3 and ALK6 in Figure 4 was confirmed by RT-PCR.

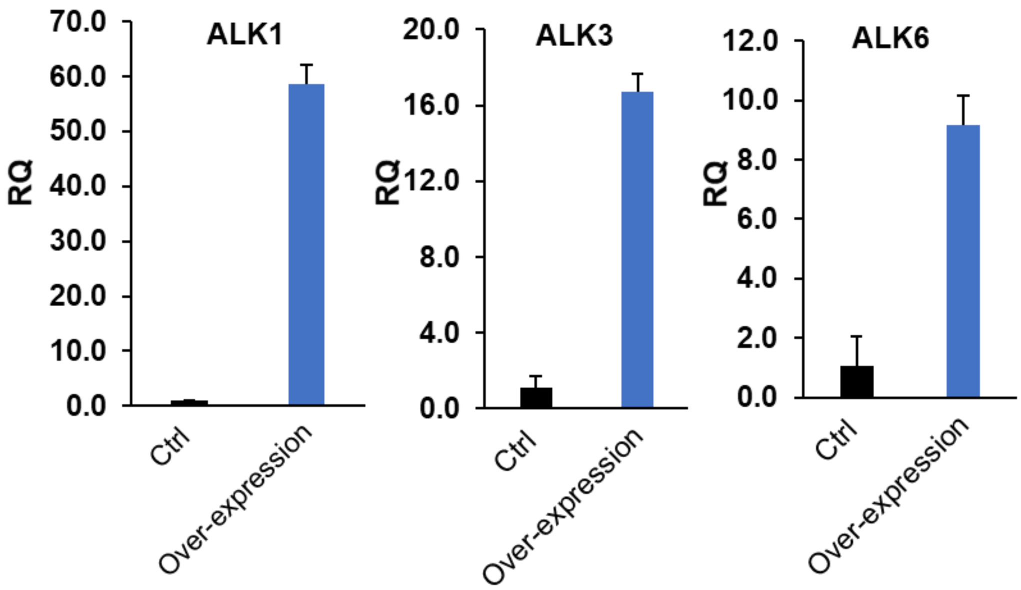

Supplementary Figure 3: Overexpression of kinase-deficient ActR-IIA^{K219R}, ActR-IIB^{K217R} and BMPR-2^{K230R} significantly down-regulated activin-A-induced BMP signaling via ALK2^{WT} upon FKBP12 dissociation in C2C12BRA cells as well.

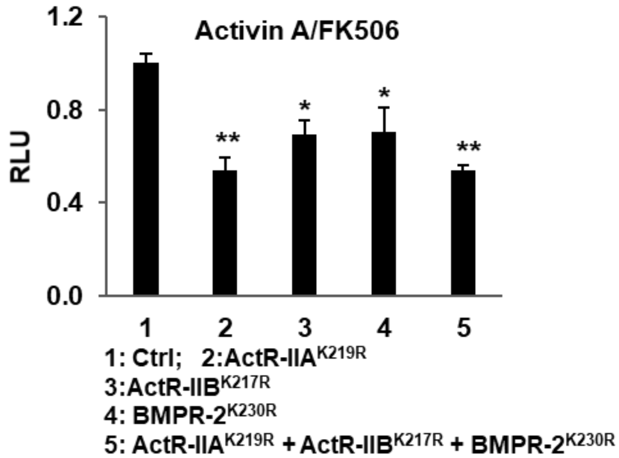

Supplement: Supplementary file 1 [file ijms-21-06498-s001.pdf]
